# Impact of mobile health interventions for mental health literacy: A systematic review and meta-analysis of randomized controlled trials

**DOI:** 10.1371/journal.pdig.0001688

**Published:** 2026-09-11

**Authors:** Jamin Patel, Sheriff Ibrahim, Tarun Reddy Katapally

**Affiliations:** 1 DEPtH Lab, School of Health Studies, Faculty of Health Sciences, Western University, London, Ontario, Canada; 2 Department of Epidemiology and Biostatistics, Schulich School of Medicine and Dentistry, Western University, London, Ontario, Canada; 3 Children’s Health Research Institute, Lawson Health Research Institute, London, Ontario, Canada; 4 Heidelberg Institute of Global Health, Heidelberg University, Heidelberg, Germany; Shanghai Mental Health Center, CHINA

## Abstract

Mental health literacy (MHL) – the ability to recognize, manage, and prevent mental health problems – is a key determinant of mental health. Mobile health (mHealth) interventions, delivered via portable digital devices such as smartphones, offer scalable, interactive, and user-tailored approaches to improve MHL. This study aimed to evaluate the impact of mHealth interventions on MHL outcomes, compared to control conditions (non-mHealth interventions, waitlist, or no intervention). This systematic review and meta-analysis searched five databases for randomized controlled trials published between January 1, 2010, and April 18, 2025. Eligible studies enrolled participants aged 12 and older and compared mHealth interventions to control groups. Two reviewers independently screened articles, extracted data, and assessed risk of bias using the Cochrane RoB 2 tool. The primary outcome was MHL, assessed using standardized mean differences (SMD) for overall MHL and its components: knowledge, help-seeking, and stigma. A random-effects meta-analysis was conducted. Out of 5,043 records identified, 14 RCTs met all inclusion criteria, encompassing 4,314 participants. mHealth interventions showed higher overall MHL (SMD = 0.13, 95% CI: 0.03–0.23), knowledge (SMD = 0.23, 95% CI: 0.09–0.37), and help-seeking (SMD = 0.26, 95% CI: 0.04–0.47; p = 0.019), compared to controls. High heterogeneity was detected (I² = 93.6%; τ² = 0.03; p < 0.001). Two studies were rated at high risk of bias; the remainder had at least some concerns. mHealth interventions modestly improved MHL outcomes, particularly knowledge and help-seeking. Although the effect sizes were small and heterogeneity was high, these interventions may offer meaningful public health value when delivered at scale.

## Author summary

Mental health literacy (MHL) – the ability to recognize, manage, and prevent mental health problems – is a key determinant of mental health. Mobile health interventions, delivered via smartphones and other portable devices, offer scalable and tailored approaches to improve MHL. This systematic review and meta-analysis evaluated randomized controlled trials comparing mHealth interventions to control conditions among participants aged 12 and older from January 2010 to April 2025. Fourteen studies (n = 4,314 participants) met the inclusion criteria. Mobile health interventions were associated with significantly higher overall MHL, knowledge, and help-seeking compared to controls. Two studies were deemed to have a high risk of bias, while others had some concerns. Despite small effect sizes and heterogeneity, mobile health interventions may provide meaningful public health benefits if delivered at scale. However, the modest effect sizes highlight the need for further improvement in the design of mHealth interventions. As the evidence base grows, meta-regression could not only help better understand between-study variability but also clarify the conditions under which mHealth interventions are most effective to inform widespread implementation.

## 1. Introduction

Mental health is an important aspect of overall well-being – it contributes to how individuals think, feel, and behave [1]. When mental health is compromised, it can result in the development of mental disorders, which involve impairments in cognition, behaviour, and the regulation of emotions [2]. Globally, mental disorders are widespread, with point prevalence estimates from the World Health Organization indicating that approximately one in eight individuals were living with a mental disorder in 2019 [2]. Mental disorders also contribute significantly to the global disease burden [3] – they are associated with higher years lived with disability, placing strain on health systems, social services, and economies worldwide [4–7].

Mental health literacy (MHL) is an important determinant of mental health that can help mitigate some of the burdens of mental disorders [8,9]. MHL includes the knowledge and attitudes related to mental health that can help recognize, manage, and prevent mental disorders, [10,11] and is distinct from mental health treatment in that it focuses on early identification rather than clinical management. Higher levels of MHL involve less aversion to help-seeking, lower levels of stigma, and greater awareness of available mental health services [12–15]. In contrast, low MHL has been associated with poor mental health outcomes. For instance, one study in Sweden found greater self-stigma toward help-seeking among individuals with lower MHL [16]. Similarly, research among older adults in China identified associations between low MHL and symptoms of anxiety and depression [17]. MHL remains low for many individuals, despite its importance for promoting mental health. A study conducted in the United States found that fewer than 50% of participants could correctly identify symptoms of depression [18]. Additionally, a Canadian report found that only 37% of Canadians felt very confident in recognizing poor mental health [19].

A promising approach to improve MHL involves the use of mobile health (mHealth) interventions, which leverage portable digital devices such as smartphones and smartwatches to deliver health services and information [20]. In this review, mHealth was operationally defined by its delivery through a portable personal device rather than by the format of its content. Accordingly, text, video, e-learning, messaging, and web-based content were considered mHealth when delivered through or specifically designed for smartphones, tablets, wearables, or similar mobile devices. The same content formats were considered broader digital interventions when delivered only through desktop computers, fixed-location systems, or stand-alone non-mobile internet platforms. While mHealth interventions have primarily been used to support public health goals, including remote health monitoring, virtual care, and disease management, [21,22] they are increasingly being adapted to enhance MHL. For instance, the Headspace platform educates individuals on techniques for guided meditation and mindfulness exercises, which have reduced stress and improved mental health [23]. Similarly, the MoodMission intervention provides users with personalized strategies to manage low mood and depression, with evidence of significant improvements in mental health outcomes [24]. The effectiveness of mHealth interventions has been evaluated in previous randomized controlled trials (RCTs), which compare intervention groups to control conditions such as waitlists or standard care [25–28]. Despite the methodological rigour of well-conducted RCTs, a single study is often insufficient to draw reliable or generalizable conclusions because it may be limited by issues such as sampling variability and random error, which limit the generalizability of its findings [29].

Systematic reviews and meta-analyses address these limitations by synthesizing findings across multiple studies [30]. However, existing reviews have synthesized mHealth tools alongside broader digital interventions, such as web-based programs and telemedicine, without focusing exclusively on RCTs [28,31–34]. For instance, Yeo et al. (2024) conducted a meta-analysis of digital interventions to improve MHL but did not report results specific to mHealth platforms, which are generally more portable, accessible, and widely adopted than other digital tools [28].

Similarly, Chen et al. (2024) included broader internet-based formats, such as video content, e-learning modules, and mental health e-cards [31]. In the present review, these content formats were considered mHealth only when delivered through a portable mobile device. Ito-Jaeger et al. (2022) included video interventions across delivery platforms and therefore did not isolate mobile-device-based interventions in its analysis [32]. Although Tian et al. (2024) examined app- and web-based tools, their scoping review lacked the quantitative synthesis offered by meta-analysis [34].

Given the proliferation of mobile technologies and the growing integration of mHealth in mental health promotion, [35] a focused meta-analysis of RCTs is both timely and necessary to inform evidence-based policy and practice. To address these gaps, this systematic review and meta-analysis synthesizes evidence from RCTs to evaluate the effectiveness of mHealth platforms in improving MHL among individuals aged 12 and older.

## 2. Methodology

This study is a systematic review and meta-analysis of RCTs evaluating mHealth interventions for improving MHL. The review protocol was pre-registered with the Open Science Framework (https://doi.org/10.17605/OSF.IO/WMX3H). The review adhered to the PRISMA 2020 reporting guidelines (S1 File), as well as the Cochrane Handbook for Systematic Reviews of Interventions.

### 2.1. Search strategy and selection criteria

The search strategy was developed with a health sciences librarian at Western University and executed on April 18, 2025. Five electronic databases were searched: MEDLINE, Embase, APA PsycInfo, CENTRAL, and Web of Science. The strategy combined terms related to mHealth, MHL, and RCTs. Keywords were drawn from prior systematic reviews and tailored to each database [22,28]. The complete search terms for MEDLINE are available in Table A in S1 Appendix. We applied no language or geographical restrictions and did not search for grey literature or trial registries. We did not seek unpublished datasets or individual participant data beyond what was reported in the published manuscripts. However, when required post-intervention summary statistics (means, standard deviations, and sample sizes) were not reported, authors were contacted to request these specific data. Only summary-level data were extracted; individual participant data were not sought.

Studies were eligible if they were RCTs published between January 1, 2010, and April 18, 2025. The 2010 cutoff was selected a priori to focus the review on the contemporary app-enabled smartphone era and to improve comparability with mobile platforms currently used in practice. Including earlier studies would have introduced interventions developed for substantially different mobile technologies and delivery environments.

Eligible studies included participants aged 12 years or older, reflecting the average age of first smartphone ownership [36]. This threshold allowed the inclusion of both adolescents and adults, reflecting the broad population targeted by many mHealth interventions and enabling exploration of potential age-related differences in intervention effectiveness. Studies had to evaluate an mHealth intervention delivered via portable digital devices (e.g., smartphones, tablets, wearables) and compare it to a control condition, such as no intervention, waitlist, or a non-mHealth comparator (e.g., face-to-face therapy, printed self-help materials). Digital tools delivered via stationary or fixed-location systems (e.g., desktop-based telehealth or hospital-based electronic health records) were excluded. Web-based applications were included only when accessed via portable personal devices such as smartphones or tablets; desktop-only or stationary digital systems were excluded. To be included in the meta-analysis, studies were required to report continuous outcome measures related to MHL – either overall MHL scores or at least one of its three core domains: mental health knowledge, help-seeking, or stigma reduction, as conceptualized by Jorm et al.[37,38] Studies that did not include MHL-related outcomes or used only categorical measures were excluded.

### 2.2. Screening

After removing duplicates in Covidence, titles and abstracts were independently screened by two reviewers (JP and NE). Studies were included if they were RCTs involving an mHealth intervention and a control group. Full texts of potentially eligible studies were then reviewed against the inclusion criteria. Disagreements were resolved through discussion; a third reviewer (SI) addressed any unresolved conflicts. If multiple publications reported data from the same trial, the most complete and relevant version was used to avoid duplication.

Two reviewers independently extracted data between April and May 2025, including publication year, country, sample size, study design (e.g., parallel, crossover), participant demographics (mean age and type of population), and baseline mental health status. Intervention details (e.g., app type, features, delivery platform) and control conditions were documented, along with outcome data (means, standard deviations, and sample sizes at the latest follow-up). Preference was given to intention-to-treat analyses. Attrition rates were extracted as the percentage of participants lost to follow-up. Adverse events were not reported in any of the included trials and were therefore not extracted.

### 2.3. Risk-of-bias assessment

The Cochrane Risk of Bias 2 (RoB 2) tool was used by two independent reviewers (JP and NE) to assess risk of bias across five domains: bias related to the randomization process; bias resulting from deviations from intended interventions; bias caused by missing outcome data; bias in how outcomes were measured; and bias in the selection of reported results. Adapted versions of RoB 2 were applied for cluster and crossover RCTs. An overall risk-of-bias judgement was generated for each study using Cochrane’s algorithms. A third reviewer (SI) resolved disagreements between the primary reviewers.

### 2.4. Statistical analysis

All analyses were conducted using the metafor package in R (version 4.5.0). Effect sizes were calculated using Hedges’ g, which adjusts for small sample bias. Hedges’ g was used to estimate standardized mean differences (SMDs) and correct for upward bias in small samples. When studies reported multiple outcomes representing different domains of MHL (e.g., knowledge, help-seeking, or stigma) but did not provide a single overall MHL score, a composite effect size was calculated. Individual SMDs (Hedges’ g) were first calculated for each outcome within the study and then combined using inverse-variance weighting to generate a single composite effect size. Each outcome was weighted by the inverse of its squared standard error to account for differences in precision across measures. When necessary, composite group means and standard deviations were also calculated using weighted averages for intervention and control groups to ensure consistency across studies reporting multiple related outcomes. For missing data (i.e., in cases where SDs were missing), they were derived from reported statistics using Cochrane-recommended transformations. All transformations and corresponding formulas are documented in the Fig M in S1 Appendix.

For stigma outcomes, values were reversed to ensure that higher SMDs consistently reflected improved MHL. All meta-analyses used random-effects models to account for between-study heterogeneity. Heterogeneity was quantified using the I² statistic, with thresholds of 25%, 50%, and 75% representing low, moderate, and high heterogeneity, respectively. Leave-one-out sensitivity analyses were conducted to assess the influence of each study on pooled estimates. Additional sensitivity analyses were conducted to test robustness by excluding studies targeting clinical or niche populations, using active controls, or employing cluster-randomized or crossover designs. Publication bias was assessed using funnel plots and Egger’s test. All analyses were stratified by MHL domain (knowledge, help-seeking, stigma), age group (12–24 years vs ≥ 25 years), and follow-up duration (≤3 months vs > 3 months). Subgroup analyses were descriptive and exploratory.

## 3. Results

### 3.1. Study selection

**Fig 1:**
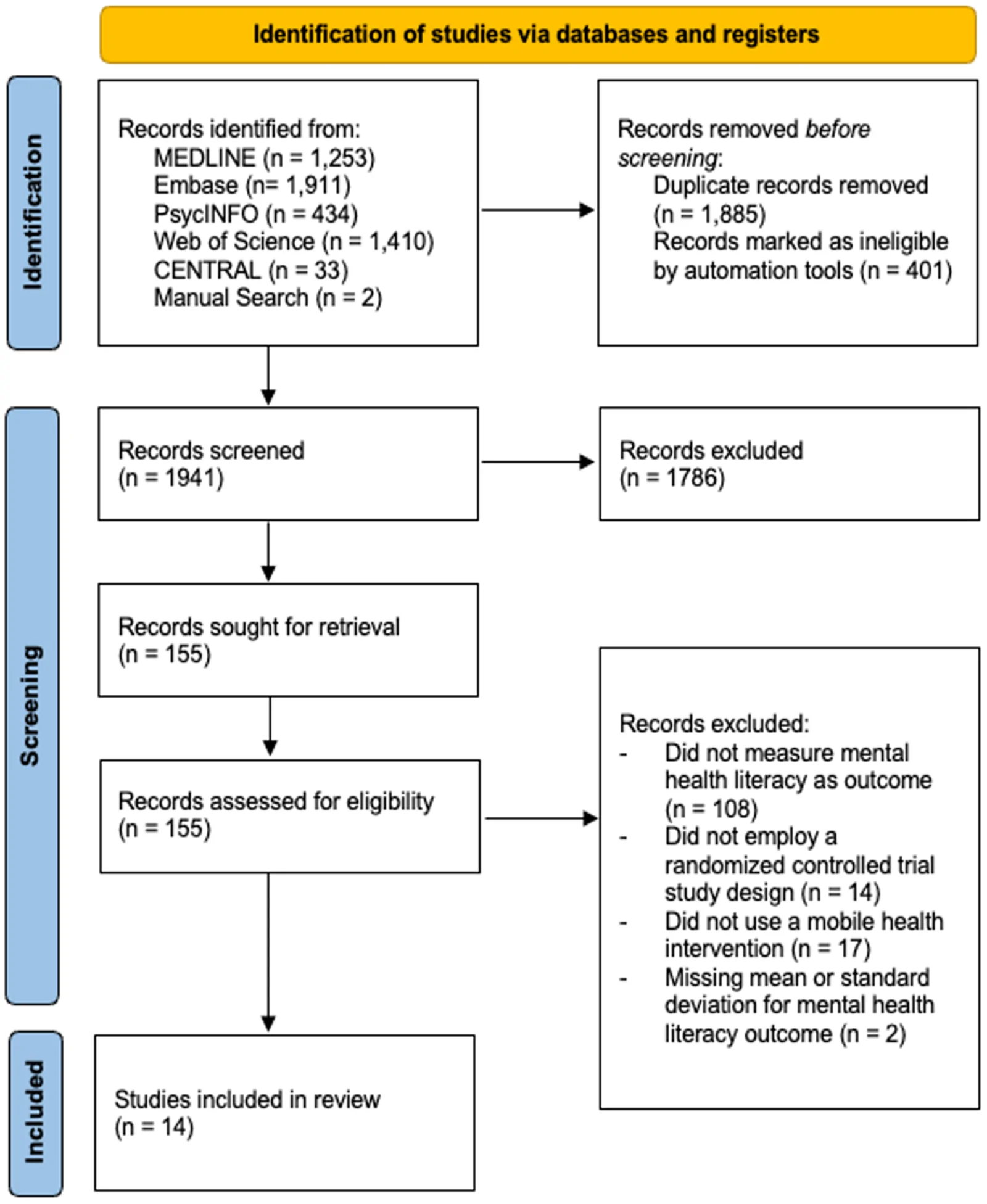
PRISMA flow diagram of study selection. Flow diagram illustrating the identification, screening, eligibility assessment, and inclusion of studies in the systematic review and meta-analysis.

A total of 5,043 records were identified through the database search (Fig 1). Two additional records were identified through manual reference list searching of a recent meta-analysis [28]. After removing 1,885 duplicates and 401 non-randomized studies through Covidence’s automation tools, 1,941 records remained for title and abstract screening. Of these, 155 articles were selected for full-text screening. Following full-text assessment, 141 articles were excluded for the following reasons: MHL not measured as an outcome (n = 108), study not designed as an RCT (n = 14), or intervention not classified as mHealth (n = 17). Two additional studies met the inclusion criteria but were excluded due to missing data on post-intervention means, standard deviations, and sample sizes; efforts to contact the authors were unsuccessful. In total, 14 RCTs, comprising 4,314 participants, were included in both the systematic review and the meta-analysis.

### 3.2. Study characteristics

The 14 RCTs included in this review were published between 2011 and 2024, with most studies (n = 12) published from 2016 onward. The full study characteristics table can be found in Table B in S1 Appendix. All studies were conducted in high-income countries, including Australia (n = 5), the United States (n = 2), Germany (n = 2), and one each in Spain, New Zealand, Norway, South Korea, and the Netherlands. Thirteen studies used a parallel-group design, while one employed a crossover design. Ten studies used individual randomization, and four used cluster randomization. Across all studies, the mean participant age was 29.8 years (SD = 10.5). Six studies targeted youth aged 12–24 years, while the remaining eight studies focused on adults aged 25 and older. Within the adult subgroup, study-arm mean ages ranged from approximately 27.5 to 42.8 years. No included trial specifically targeted adults aged 65 years or older, and older-adult-specific outcome estimates were not available. The studies assessed a wide range of populations, including individuals with self-reported depression, clinically relevant internalizing symptoms, non-suicidal self-injury, postpartum mothers, office workers, construction workers, and Syrian refugees.

Twelve of the included interventions were delivered through smartphone or web applications. One study used a text-messaging platform, and another used a mobile game-based application [39,40]. The digital platforms evaluated included MoodKit, MoodPrism, MoodMission, MoodHacker, Mind your Mate, MyTeen, esTOCma, MindDoc, MATESmobile, TalkLife, MoodGYM, WeClick, Sanadak, Happy Mother App, and Moving Stories (Table A in S1 Appendix). Key features of the interventions are summarized in the Table C in S1 Appendix. Educational media – defined as psychoeducational content delivered through formats such as text modules, videos, or interactive learning materials – was the most common feature, included in 11 interventions [24,27,40–48]. Mood-tracking, defined as tools that allow users to record or monitor emotional states over time through self-reported ratings or logs, was present in seven interventions [24,27,41,42,44,45,48]. Personalization, referring to features that tailor content or feedback based on individual responses or usage patterns, appeared in six interventions [13,24,27,42,44,48]. Cognitive-behavioural therapy (CBT) components – structured techniques derived from CBT such as cognitive restructuring or behavioural exercises – were included in five interventions [27,39,41,47,48]. Gamification, defined as the use of game-like elements such as points, rewards, or challenges to enhance engagement, was the least frequent feature and appeared in four interventions [42,43,48,49].

Comparator conditions included waitlist (n = 6), no intervention (n = 1), and active comparators (n = 7), such as web-based psychoeducation, standard care, and in-person programs. MHL outcomes were assessed across four domains: overall MHL (n = 4 studies), knowledge (n = 7 studies), help-seeking (n = 8 studies), and stigma reduction (n = 5 studies). Many studies assessed multiple domains using distinct instruments, resulting in 21 unique outcome measures. Commonly used tools included the Mental Health Literacy Questionnaire, the General Help-Seeking Questionnaire, and the Depression Stigma Scale, although several studies used non-validated custom instruments [41,42]. Follow-up periods ranged from immediately post-intervention or 10 days to 12 months. All studies reported intention-to-treat analyses, although attrition varied considerably across trials, ranging from 5% to 92%.

### 3.3. Risk-of-bias

Risk of bias was assessed for all 14 RCTs; the traffic light plot is provided in Fig A in S1 Appendix (pg 11). Two studies were judged to be at high risk of bias overall, [27,46] while the remaining studies were rated as having some concerns. Common limitations included unclear allocation concealment, unblinded outcome assessment, and the use of self-reported or study-specific instruments without validation. Although randomization was generally reported, several studies lacked pre-registration or appropriate intention-to-treat analysis.

## 4. Meta-Analysis

### 4.1. Overall MHL

**Fig 2:**
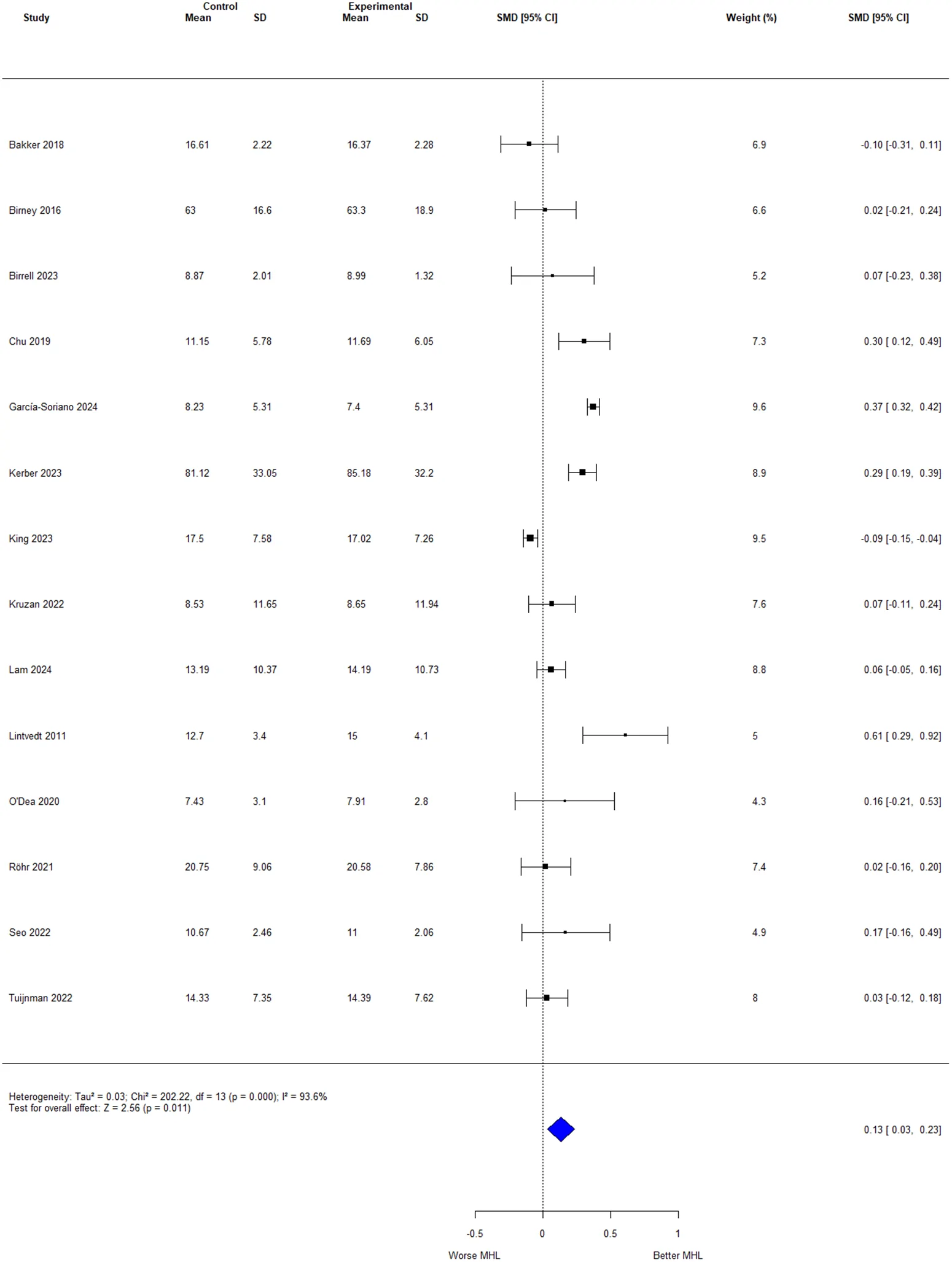
Forest plot of overall effect of mHealth interventions on mental health literacy. Forest plot displaying standardized mean differences (Hedges’ g) and 95% confidence intervals for the overall effect of mobile health interventions on mental health literacy across included randomized controlled trials. The pooled effect estimate from the random-effects model is shown.

Across all 14 RCTs, participants allocated to mHealth interventions had significantly higher MHL scores at post-intervention compared with controls (SMD = 0.13, 95% CI: 0.03–0.23; p = 0.011) (Fig 2). Heterogeneity was high (I² = 93.6%; τ² = 0.03; p < 0.001), suggesting substantial between-study variability.

### 4.2. MHL domains

**Fig 3:**
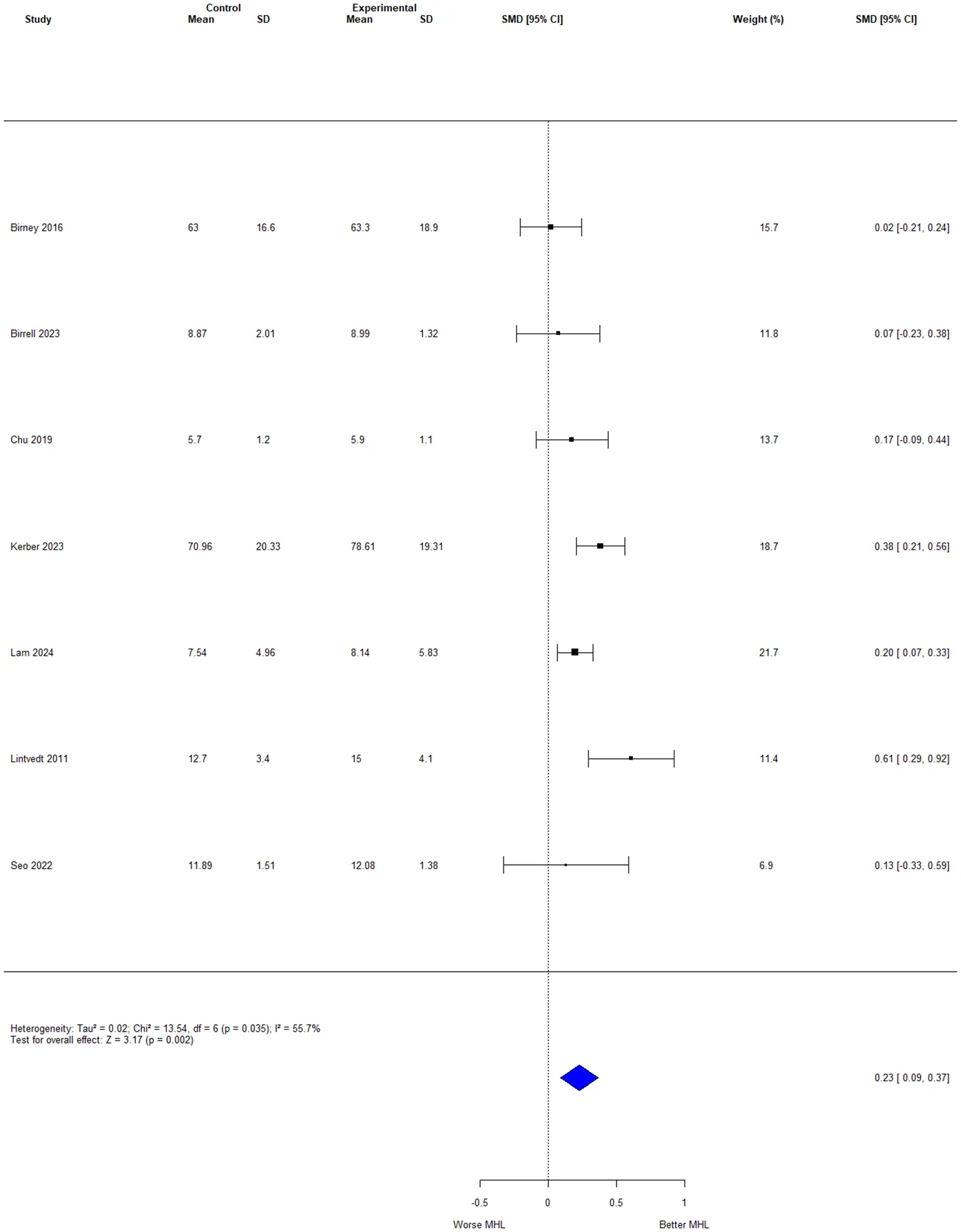
Forest plot stratified by mental health literacy domains. Forest plot presenting subgroup analyses by mental health literacy domains, including knowledge, stigma, and help-seeking. Effect sizes (Hedges’ g) with 95% confidence intervals are shown for each study and pooled within each domain.

**Fig 4:**
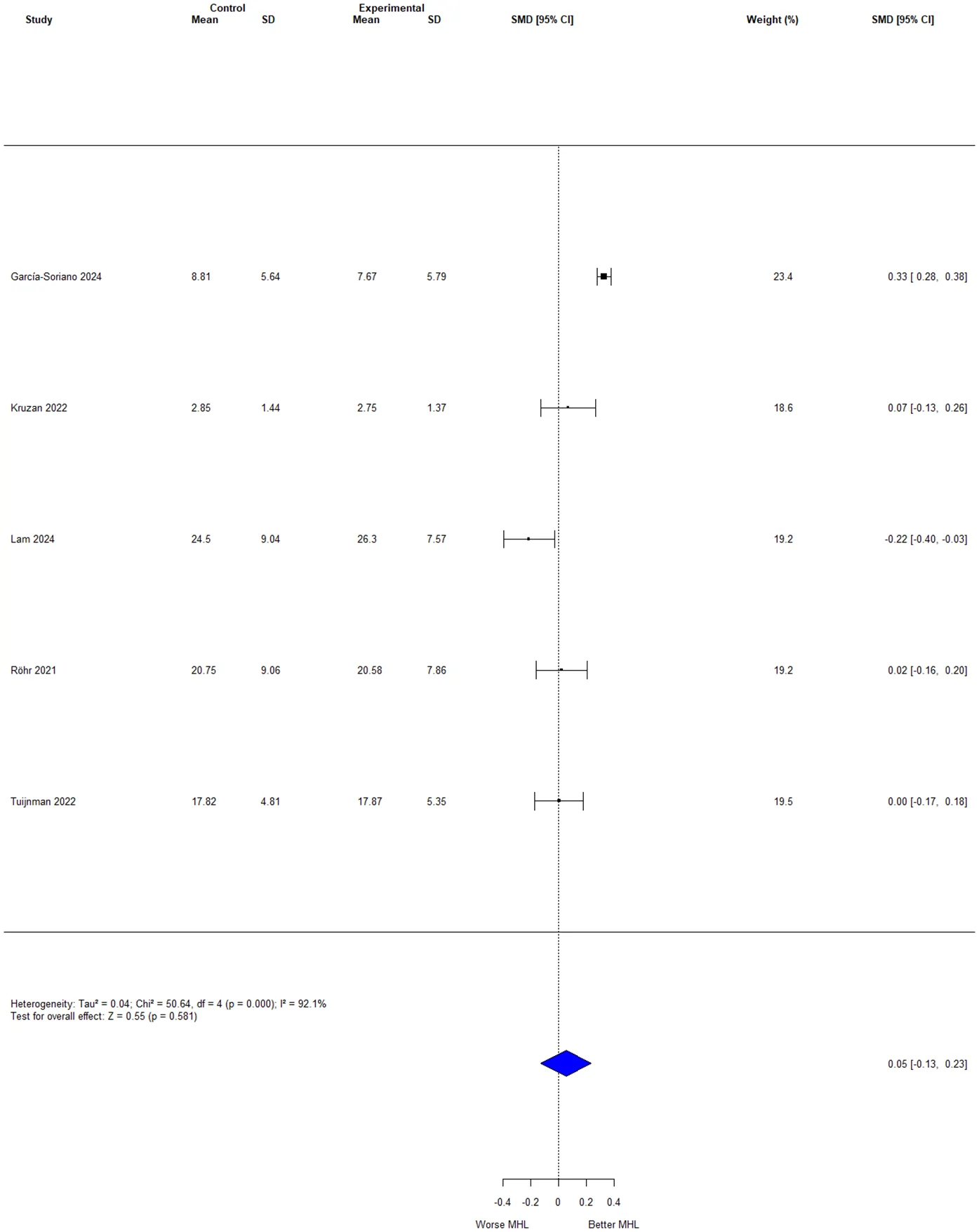
Risk of bias summary for included studies. Summary of risk of bias assessments across included randomized controlled trials using the Cochrane Risk of Bias 2 tool.

**Fig 5:**
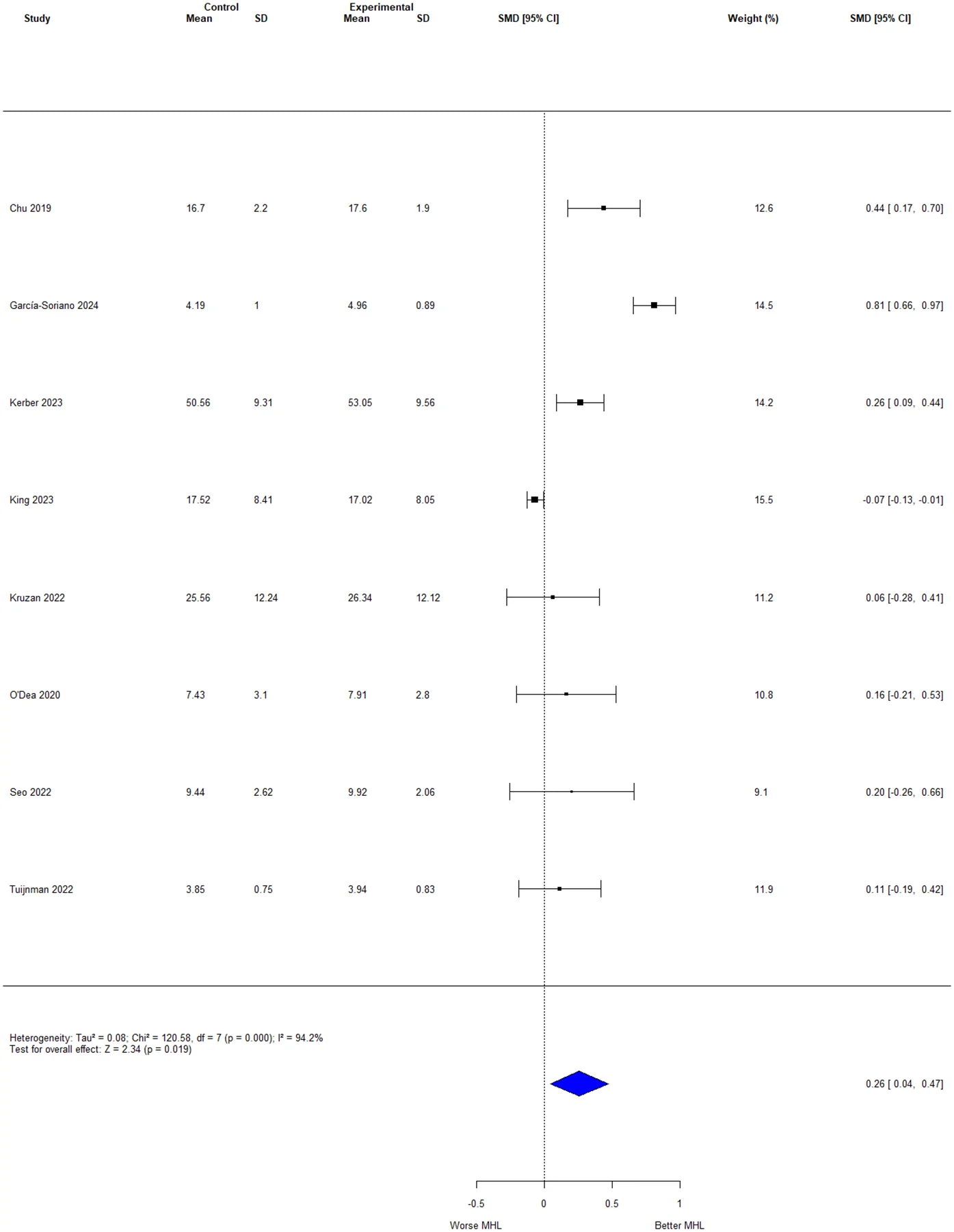
Funnel plot assessing publication bias. Funnel plot of effect sizes against standard errors for included studies to assess potential publication bias. Symmetry of the distribution is visually evaluated.

For the knowledge domain (n = 7 studies), the pooled SMD was 0.23 (95% CI: 0.09–0.37; p = 0.002), indicating significantly higher mental health knowledge scores among participants receiving mHealth interventions compared with controls, with moderate heterogeneity (I² = 55.7%; τ² = 0.02; p = 0.035) (Fig 3). For stigma-related outcomes (n = 5 studies), the pooled SMD was 0.05 (95% CI: -0.13–0.23; p = 0.581), indicating no statistically significant difference between mHealth and control groups, with high heterogeneity (I² = 92.1%; τ² = 0.04; p < 0.001) (Fig 4). For help-seeking outcomes (n = 8 studies), the pooled SMD was 0.26 (95% CI: 0.04–0.47; p = 0.019), indicating a statistically significant difference favouring mHealth interventions, with high heterogeneity (I² = 94.2%; τ² = 0.08; p < 0.001) (Fig 5).

### 4.3. Subgroup analyses

As shown in Fig C to Fig F in S1 Appendix, subgroup analyses were performed to explore differences across age groups and follow-up duration. Among youth (aged 12–24 years), six studies were included. The pooled SMD was 0.19 (95% CI: 0.03– 0.35; p = 0.021), with moderate heterogeneity (I² = 65.1%; τ² = 0.030; p = 0.014). For adults aged 25 and older (n = 8 studies), the pooled SMD was 0.10 (95% CI: -0.04–0.23; p = 0.152), indicating no statistically significant effect, with substantial heterogeneity (I² = 96.3%; τ² = 0.03; p < 0.001). In analyses stratified by follow-up time, eight studies with short-term follow-up (≤3 months) produced a pooled SMD of 0.14 (95% CI: -0.02–0.30; p = 0.085), with high heterogeneity (I² = 96.3%; τ² = 0.05; p < 0.001). Six studies with longer follow-up durations (>3 months) showed a significant effect (SMD = 0.13; 95% CI: 0.01–0.25; p = 0.037), with moderate heterogeneity (I² = 57.1%; τ² = 0.010; p = 0.040).

### 4.4. Publication Bias

There was no clear visual indication of publication bias or small-study effects based on the funnel plot as shown in the Fig L in S1 Appendix. Additionally, Egger’s regression test indicated no statistically significant funnel plot asymmetry (t = -0.437, df = 12, p = 0.670). However, publication bias could not be definitively ruled out due to the limited number of studies included in this review.

### 4.5. Sensitivity analysis

Excluding studies with high risk of bias, clinical or niche populations, active control groups, or cluster-randomized and crossover designs did not substantially change the pooled estimates as highlighted in Fig F to Fig K in S1 Appendix. A leave-one-out sensitivity analysis was also conducted to assess the impact of individual studies on the pooled effect estimate and heterogeneity, as shown in Table D in S1 Appendix. Across the 14 iterations, the pooled SMD ranged from 0.104 to 0.156. However, the overall direction and approximate magnitude of the effect remained consistent. Between-study heterogeneity ranged from 78.9% to 89.4%, indicating moderate variability in heterogeneity across iterations, but no substantive deviation from the original model.

## 5. Discussion

### 5.1. Overall findings

This systematic review and meta-analysis of RCTs assessed the impact of mHealth interventions on MHL, compared to control conditions (i.e., no intervention, waitlist, or non-mHealth comparators). Fourteen RCTs, involving 4,314 participants, were included in this review. Although the included studies were rated as having some concerns or high risk of bias, the overall findings suggest that participants receiving mHealth interventions demonstrated higher MHL scores compared to those allocated to the control conditions.

These findings are consistent with previous meta-analyses evaluating digital interventions for MHL, which have similarly demonstrated beneficial effects [28,37]. However, prior reviews did not isolate mHealth interventions. The distinction between mHealth interventions and other digital health interventions is important because mHealth enables unique functionalities that may enhance MHL through mechanisms distinct from other digital modalities [22,50]. For instance, six studies included in this review integrated real-time mood-tracking of daily behaviours and emotional states through smartphone applications, leveraging the portability and constant connectivity of mobile devices to identify psychological changes as they occurred and deliver timely resources [24,27,41,42,44,48]. Moreover, all interventions in this review were deployed on participants’ smartphones, allowing individuals to exercise greater control over their learning and fostering autonomy [51,52]. By enabling access across everyday contexts such as home, commutes, and school, mHealth interventions reduce dependence on fixed locations, scheduled sessions, and specialized equipment [53,54].

Although mHealth interventions were associated with higher MHL scores compared to control groups, the small magnitude of effect may reflect the limited functionality of many current intervention designs. In this review, fewer than half of the interventions incorporated personalized content, with most relying primarily on static content delivery rather than fully interactive or adaptive features. Personalized content referred to recommendations or feedback tailored to an individual’s responses, such as suggested coping activities based on a reported mood. Interactive features required active user input or two-way engagement, such as mood logs, self-assessments, quizzes, cognitive-behavioural exercises, or peer communication. Adaptive features referred to systems that automatically modified the content, sequence, timing, or intensity of the intervention in response to accumulating user data or engagement patterns; such functionality was uncommon among the included interventions. As a result, the educational potential of mobile platforms may not have been fully utilized. Emerging approaches, including predictive and generative artificial intelligence, offer new pathways to enhance personalization and responsiveness, [50,55] potentially addressing the limitations of current mHealth interventions for MHL.

One promising approach to enhance current digital MHL interventions is the use of predictive models, which can analyze patterns in self-reported and passively collected data such as sleep, mood, smartphone usage, and geolocation to provide real-time, tailored recommendations [56–58]. These artificial intelligence-enabled systems can adapt intervention content based on user behaviour and engagement patterns, enabling more personalized delivery of mental health information and resources. For instance, machine learning models applied to digital phenotyping data have demonstrated the ability to identify early indicators of mental health changes, which can help deliver targeted prompts or educational resources through mobile platforms [56,59]. Such approaches may enhance MHL by providing users with context-specific insights and timely guidance, thereby improving awareness of symptoms, available resources, and opportunities for help-seeking.

In addition to synthesizing overall MHL effects, this review separately examined the impact of mHealth interventions on individual MHL domains, an approach undertaken by previous meta-analyses [31,37]. These results support the hypothesis that mHealth, as a distinct class of digital health interventions, can facilitate gains in MHL when compared to standard or inactive comparators.

Sensitivity analyses provided additional context for interpreting the modest effect sizes observed in this review. In particular, when studies with active comparator conditions were excluded, the pooled effect estimates for overall MHL increased. This pattern suggests that the relative impact of mHealth interventions may appear smaller when compared with other structured mental health programs, such as psychoeducational or in-person interventions, which may themselves improve MHL. When compared with minimal or inactive control conditions, however, mHealth interventions appear to demonstrate stronger relative effects. These findings highlight the potential value of mHealth interventions as scalable tools for improving MHL, particularly in settings where traditional mental health education or services may be less accessible.

### 5.2. Knowledge

The finding that mHealth interventions improved mental health knowledge more than control conditions is consistent with previous studies, which also reported positive effects. However, in this review, the effect size was small, whereas prior meta-analyses by Chen et al. (2024) and Nazari et al. (2023) reported moderate to large effects [31,37]. One possible explanation for this discrepancy is that this review focused exclusively on mHealth interventions, which are particularly vulnerable to distractions from notifications, multitasking, and environmental interruptions, [60,61] potentially limiting knowledge acquisition. The Cognitive Theory of Multimedia Learning posits that individuals learn more effectively when exposed to information through multiple channels (text, audio, and visuals), particularly when content is interactive [62]. mHealth interventions frequently leverage these strategies through videos, interactive prompts requiring users to periodically enter information, and opportunities for two-way communication between providers and others in their support networks [63,64]. Additionally, the portable format of mHealth interventions allows for flexible, repeated engagement, which may reinforce learning over time [65]. Moreover, personalization features, common in many mHealth apps, are supported by evidence showing that tailored information improves learning more effectively than generic content [66]. As the evidence base expands, it will be important to examine whether more interactive and personalized formats contribute to greater improvements in mental health knowledge.

### 5.3. Stigma reduction

Although mHealth interventions showed higher overall MHL and mental health knowledge, the results did not show a significant difference in mental health stigma reduction. This finding aligns with Nazari et al.’s (2023) meta-analysis, which reported no significant effects of web-based interventions on reducing stigma [37]. While Nazari et al. focused on web-based interventions rather than mobile platforms specifically, the comparison provides useful context because both approaches fall within the broader category of digital MHL interventions. In contrast, Chen et al. (2024) reported modest improvements in stigma following internet-based interventions [31]. One possible explanation for this discrepancy is that Chen et al. included a broader range of digital tools, such as multimedia campaigns and structured psychoeducational programs, which may have more explicitly targeted stigma-related beliefs.

The absence of significant stigma effects in this review may also reflect the specific content of the mHealth interventions included in the analysis. While several interventions incorporated educational materials aimed at improving mental health knowledge or symptom recognition, relatively few explicitly targeted stigma-related beliefs or attitudes [24,27,40–48]. As a result, the intervention content may have been better suited to improving knowledge and help-seeking behaviours than to altering deeply rooted perceptions about mental illness. Reducing mental health stigma often requires interventions that directly address social norms, cultural narratives, and interpersonal attitudes, which was not the primary focus of many of the mHealth interventions included in this review [67–69].

Reducing mental health stigma often requires sustained exposure to interventions that address social norms, cultural narratives, and interpersonal attitudes [67]. Stigma is frequently reinforced by societal and cultural values and may become internalized over time, making it more difficult to change through brief or standardized digital interventions [68,70]. In addition, mobile applications may lack the cultural tailoring or social interaction components necessary to effectively challenge stigma in diverse populations. To enhance the potential for mHealth interventions to reduce stigma, future research should focus on integrating culturally sensitive and stigma-focused content into mobile platforms, including interventions designed specifically to challenge stigmatizing beliefs and promote more supportive attitudes toward mental health [71].

### 5.4. Help-seeking

Although no significant improvements were observed in stigma reduction, small differences were found between the mHealth intervention and control conditions for help-seeking. This finding is consistent with Chen et al.’s (2024) meta-analysis, which also reported modest gains in help-seeking following internet-based MHL interventions [31]. In contrast, Nazari et al. (2023) found no significant improvements in help-seeking following web-based MHL interventions, [37] although their analysis was characterized by substantial heterogeneity in the help-seeking model, which may have contributed to the null findings. The modest differences observed in this review may be partly explained by specific features of mHealth interventions that promote greater autonomy and control over the help-seeking process. According to the Theory of Planned Behaviour, perceived behavioural control is a key determinant of help-seeking behaviour [72]. mHealth interventions may enhance this sense of control by providing users with direct access to mental health services, self-assessment tools, and localized care resources, thereby addressing logistical barriers that often impede help-seeking [73]. Future research should examine the relative contribution of these intervention components – such as direct service access, private self-assessment, and real-time support tools – to identify which strategies are most effective in promoting sustained help-seeking behaviours.

### 5.5. Age-stratified subgroup analysis

In addition to examining effects across various mHealth domains, age-stratified subgroup analyses revealed a significant effect of mHealth interventions on MHL in youth aged 12–24 years, whereas no significant effect was observed among adults aged 25 years and older. However, the 25 years and older category should not be interpreted as representative of older adults, because the included adult samples were predominantly composed of younger and middle-aged adults. The absence of a significant pooled effect in this subgroup therefore does not establish that mHealth interventions are ineffective among older adults.

Similar to this study, Nazari et al. (2023) reported improvements in MHL among youth following web-based interventions, supporting the finding that younger populations may derive greater benefit from digital health approaches [37]. Adolescents and young adults typically demonstrate higher digital literacy and greater comfort navigating mobile platforms, which may facilitate more active engagement with mHealth content [74,75]. In addition, youth are often in critical developmental periods characterized by openness to new information, identity formation, and evolving health behaviours, which may render them more responsive to interventions targeting MHL [76,77]. For older adults, the potential benefits of portable access may be offset by barriers related to digital literacy, accessibility, device access, privacy concerns, and usability. Age-inclusive design – including simplified navigation, readable content, technical support, and alternatives to text-heavy interactions – may therefore be necessary to support equitable uptake [78]. However, due to the limited number of studies, formal statistical tests comparing effects across age groups could not be conducted [79]. Future research will be needed to verify whether true differences exist as additional evidence accumulates.

### 5.6. Follow-up time stratification

Another key contribution of this review to the current literature is its attention to follow-up duration, a gap left unaddressed in earlier work. Consistent with previous reviews, which found that improvements in knowledge and attitudes were more commonly observed after at least four weeks of follow-up, [80] the present review found that mHealth interventions contributed to significant differences in MHL between the mHealth intervention and control groups, only when outcomes were assessed beyond three months. This time-dependent pattern also aligns with evidence suggesting that cognitive and behavioural changes may require iterative exposure and longer durations to emerge [81]. Although Chen et al. (2024) partially addressed the timing gap by analyzing MHL outcomes across multiple follow-up points after internet-based interventions (ranging from one week to two years), their meta-analysis did not calculate a composite MHL score to enable a more comprehensive evaluation of the overall impact of mHealth interventions on MHL [31]. In contrast, the present review incorporated a composite score (overall MHL) to provide a more comprehensive evaluation of the impact of mHealth interventions on MHL.

### 5.7. Strengths and limitations

While this review contributes novel insights by systematically evaluating RCTs of mHealth interventions aimed at improving MHL, several methodological limitations should be considered. Despite broad eligibility criteria allowing for a range of populations, intervention formats, and outcome measures, only 14 RCTs met the final inclusion criteria. This reflected deliberate restrictions aimed at isolating mHealth-specific effects and prioritizing trials with strong internal validity. However, the limited evidence base may have constrained statistical power, increasing the likelihood of Type II error – failing to detect effects that might be present. In addition, studies that reported only categorical or non-continuous MHL outcomes were excluded from the meta-analysis because SMDs require continuous outcome measures, which may have resulted in the omission of potentially relevant studies.

Moreover, all included RCTs were conducted in high-income countries, which may limit the generalizability of these findings to low- and middle-income countries where digital infrastructure, smartphone access, and cultural contexts surrounding mental health may differ [82]. Although one trial was conducted in South Korea and one involved Syrian refugees in Germany, the evidence base remained concentrated in Western cultural contexts. Cultural differences in beliefs about mental illness, stigma, help-seeking, family roles, language, and trust in digital services may affect both engagement with and the effectiveness of mHealth interventions. Accordingly, the pooled findings may not generalize across cultural settings, and future studies should prioritize culturally adapted or co-designed interventions evaluated in diverse cultural and linguistic populations.

Additionally, although funnel plot asymmetry was not strongly suggestive of small-study effects, publication bias could not be ruled out due to the limited number of trials and variability in outcome reporting. Another limitation of this review is that although it restricted inclusion to mHealth interventions, differences in intervention features (e.g., content, intensity, engagement strategies), comparator conditions (ranging from waitlist controls to active psychoeducation), and participant populations (e.g., age groups, baseline MHL levels, cultural settings) may have contributed to high heterogeneity. As a result, pooled estimates should be interpreted with caution, recognizing that observed effects likely reflect the combined influence of multiple factors rather than the isolated impact of mHealth interventions alone.

## 6. Conclusion

This systematic review and meta-analysis synthesized RCTs evaluating mHealth interventions for MHL. The results showed small differences in overall MHL, mental health knowledge, and help-seeking compared to control conditions, demonstrating mHealth’s potential to improve MHL and potentially contribute to the broader goal of preventing mental health challenges. However, the modest effect sizes highlight the need for further improvement in the design of mHealth interventions. As the evidence base grows, meta-regression could not only help better understand between-study variability but also clarify the conditions under which mHealth interventions are most effective to inform widespread implementation.

## Supporting information

S1 File PRISMA Checklist. (DOCX)

S1 Appendix Table of Contents. Table A. Search strategies for electronic databases 2. Table B. Summary of study characteristics, including study design, setting, and populationa 6. Table C. Summary of Features Included in mHealth Interventions for MHLa 9. Table D. Leave-one-out sensitivity analysis 10. Fig A. Traffic light plot showing results of the risk-of-bias assessment across studies 11. Fig B. Traffic light plot showing results of the risk-of-bias assessment across studies 11. Fig C. Forest plot of meta-analysis showing the effect of mHealth interventions on MHL among youth (aged 12–24 years) 12. Fig D. Forest plot of meta-analysis showing the effect of mHealth interventions on MHL among adults (aged older than 25 years) 13. Fig E. Forest plot of meta-analysis showing the effect of mHealth interventions on MHL among studies with short-term follow-up (≤3 months) 14. Fig F. Forest plot of meta-analysis showing the effect of mHealth interventions on MHL among studies with long-term follow-up (>3 months) 15. Fig G. Overall meta-analysis excluding studies with an active control group 16. Fig H. Forest plot of overall meta-analysis excluding studies with high risk of bias 17. Fig I. Forest plot of overall meta-analysis excluding studies with a niche participant population 18. Fig J. Forest plot of overall meta-analysis excluding studies that used a cluster or cross-over experimental study design 19. Fig K. Forest plot of overall meta-analysis excluding studies that involved participants with existing mental health symptoms 20. Fig L. Funnel plot of overall meta-analysis assessing publication bias 21. Fig M. Formulas for post-intervention summary statistics and handling unit-of-analysis issues.
(DOCX)
