# Supplementary material for: Impact of mobile health interventions for mental health literacy: A systematic review and meta-analysis of randomized controlled trials
Source: PLOS Digit Health. 2026 Sep 11;5(9):e0001688. doi: 10.1371/journal.pdig.0001688 (PMC13567821; doi:10.1371/journal.pdig.0001688)
Supplement: Supplementary file 2 [file pdig.0001688.s002.docx]

**Table of Contents**

[Table A. Search strategies for electronic databases	2](#_Toc202369001)

[Table B. Summary of study characteristics, including study design, setting, and population^a^	6](#_Toc202369002)

[Table C. Summary of Features Included in mHealth Interventions for MHL^a^	9](#_Toc202369003)

[Table D. Leave-one-out sensitivity analysis	10](#_Toc202369004)

[Fig A. Traffic light plot showing results of the risk-of-bias assessment across studies	11](#_Toc202369005)

[Fig B. Traffic light plot showing results of the risk-of-bias assessment across studies	11](#_Toc202369006)

[Fig C. Forest plot of meta-analysis showing the effect of mHealth interventions on MHL among youth (aged 12–24 years)	12](#_Toc202369007)

[Fig D. Forest plot of meta-analysis showing the effect of mHealth interventions on MHL among adults (aged older than 25 years)	13](#_Toc202369008)

[Fig E. Forest plot of meta-analysis showing the effect of mHealth interventions on MHL among studies with short-term follow-up (≤3 months)	14](#_Toc202369009)

[Fig F. Forest plot of meta-analysis showing the effect of mHealth interventions on MHL among studies with long-term follow-up (>3 months)	15](#_Toc202369010)

[Fig G. Overall meta-analysis excluding studies with an active control group	16](#_Toc202369011)

[Fig H. Forest plot of overall meta-analysis excluding studies with high risk of bias	17](#_Toc202369012)

[Fig I. Forest plot of overall meta-analysis excluding studies with a niche participant population	18](#_Toc202369013)

[Fig J. Forest plot of overall meta-analysis excluding studies that used a cluster or cross-over experimental study design	19](#_Toc202369014)

[Fig K. Forest plot of overall meta-analysis excluding studies that involved participants with existing mental health symptoms	20](#_Toc202369015)

[Fig L. Funnel plot of overall meta-analysis assessing publication bias	21](#_Toc202369016)

Fig M. Formulas for post-intervention summary statistics and handling unit-of-analysis issues

# Table A. Search strategies for electronic databases

**MEDLINE**

Date of latest search: April 21, 2025

Ovid MEDLINE(R) ALL <1946 to April 18, 2025>

1	Mental Health/ed, pc, td [Education, Prevention & Control, Trends]	1301

2	Health Literacy/ or Literacy/	13323

3	("mobile health" or "mHealth" or "mobile application*" or "mobile app*" or "smartphone*" or "digital health" or "mobile device*" or "mobile technolog*" or "mobile phone*" or "tablet*" or "eHealth" or "web app*" or "app" or "apps" or "gamification" or "iPhone*" or "Android" or "digital platform*" or "mobile tool" or "digital application*" or "digital app*" or "smart device*" or "wearable device*" or "fitness tracker*" or "smartwatch*" or "Apple Watch" or "Fitbit" or "Garmin" or "Samsung Galaxy Watch" or "mobile intervention*" or "mobile platform*" or "mobile solution*" or "health app*" or "mobile intervention" or "mobile" or "smartphone" or "iOS" or "Android" or "digital mental health intervention" or "mental health app" or "digital health platform" or "electronic health platform" or "eHealth platform" or "uHealth platform" or "ubiquitous health").mp. [mp=title, book title, abstract, original title, name of substance word, subject heading word, floating sub-heading word, keyword heading word, organism supplementary concept word, protocol supplementary concept word, rare disease supplementary concept word, unique identifier, synonyms, population supplementary concept word, anatomy supplementary concept word]	330188

4	("mental health" or "mental illness*" or "mental disorder*" or "psychological health" or "emotional health" or "mental wellness" or "mental well-being" or "psychological well-being" or "psychosocial well-being" or "anxi*" or "depress*" or "behavioural disorder*" or "cognitive health" or "psychological function*" or "burnout" or "mental health condition*" or "suicid*").mp. [mp=title, book title, abstract, original title, name of substance word, subject heading word, floating sub-heading word, keyword heading word, organism supplementary concept word, protocol supplementary concept word, rare disease supplementary concept word, unique identifier, synonyms, population supplementary concept word, anatomy supplementary concept word]	1390265

5	("literacy" or "knowledge" or "education" or "awareness" or "self-efficacy" or "help-seeking" or "help-seeking" or "support-seeking" or "support seeking" or "stigma*" or "competence" or "empowerment").mp. [mp=title, book title, abstract, original title, name of substance word, subject heading word, floating sub-heading word, keyword heading word, organism supplementary concept word, protocol supplementary concept word, rare disease supplementary concept word, unique identifier, synonyms, population supplementary concept word, anatomy supplementary concept word]	2463305

6	("RCT" or "randomized controlled trial*" or "randomised controlled trial*" or "randomized clinical trial*" or "randomised clinical trial*" or "controlled clinical trial*" or "random assignment" or "randomized study" or "randomised study" or "random allocation" or "controlled trial" or "clinical trial").mp. [mp=title, book title, abstract, original title, name of substance word, subject heading word, floating sub-heading word, keyword heading word, organism supplementary concept word, protocol supplementary concept word, rare disease supplementary concept word, unique identifier, synonyms, population supplementary concept word, anatomy supplementary concept word]	1572932

7	limit 6 to yr="2010 - 2025"	880344

8	Mobile Applications/st, td [Standards, Trends]	1180

9	3 or 8	330188

10	2 or 5	2463305

11	1 or 4	1390265

12	7 and 9 and 10 and 11	1253

**APA PsycInfo <2002 to April 2025 Week 1>**

Date of latest search: April 21, 2025

1	Mental Health/ed, pc, td [Education, Prevention & Control, Trends]	0

2	Health Literacy/ or Literacy/	21086

3	("mobile health" or "mHealth" or "mobile application*" or "mobile app*" or "smartphone*" or "digital health" or "mobile device*" or "mobile technolog*" or "mobile phone*" or "tablet*" or "eHealth" or "web app*" or "app" or "apps" or "gamification" or "iPhone*" or "Android" or "digital platform*" or "mobile tool" or "digital application*" or "digital app*" or "smart device*" or "wearable device*" or "fitness tracker*" or "smartwatch*" or "Apple Watch" or "Fitbit" or "Garmin" or "Samsung Galaxy Watch" or "mobile intervention*" or "mobile platform*" or "mobile solution*" or "health app*" or "mobile intervention" or "mobile" or "smartphone" or "iOS" or "Android" or "digital mental health intervention" or "mental health app" or "digital health platform" or "electronic health platform" or "eHealth platform" or "uHealth platform" or "ubiquitous health").mp. [mp=title, abstract, heading word, table of contents, key concepts, original title, tests & measures, mesh word]	57343

4	("mental health" or "mental illness*" or "mental disorder*" or "psychological health" or "emotional health" or "mental wellness" or "mental well-being" or "psychological well-being" or "psychosocial well-being" or "anxi*" or "depress*" or "behavioural disorder*" or "cognitive health" or "psychological function*" or "burnout" or "mental health condition*" or "suicid*").mp. [mp=title, abstract, heading word, table of contents, key concepts, original title, tests & measures, mesh word]	770542

5	("literacy" or "knowledge" or "education" or "awareness" or "self-efficacy" or "help-seeking" or "help-seeking" or "support-seeking" or "support seeking" or "stigma*" or "competence" or "empowerment").mp. [mp=title, abstract, heading word, table of contents, key concepts, original title, tests & measures, mesh word]	927899

6	("RCT" or "randomized controlled trial*" or "randomised controlled trial*" or "randomized clinical trial*" or "randomised clinical trial*" or "controlled clinical trial*" or "random assignment" or "randomized study" or "randomised study" or "random allocation" or "controlled trial" or "clinical trial").mp. [mp=title, abstract, heading word, table of contents, key concepts, original title, tests & measures, mesh word]	89374

7	limit 6 to yr="2010 - 2025"	71490

8	Mobile Applications/st, td [Standards, Trends]	0

9	3 or 8	57343

10	2 or 5	927899

11	1 or 4	770542

12	7 and 9 and 10 and 11	434

**Embase Classic+Embase <1947 to 2025 April 18>**

Date of latest search: April 21, 2025

1	Mental Health/ed, pc, td [Education, Prevention & Control, Trends]	3

2	Health Literacy/ or Literacy/	32761

3	("mobile health" or "mHealth" or "mobile application*" or "mobile app*" or "smartphone*" or "digital health" or "mobile device*" or "mobile technolog*" or "mobile phone*" or "tablet*" or "eHealth" or "web app*" or "app" or "apps" or "gamification" or "iPhone*" or "Android" or "digital platform*" or "mobile tool" or "digital application*" or "digital app*" or "smart device*" or "wearable device*" or "fitness tracker*" or "smartwatch*" or "Apple Watch" or "Fitbit" or "Garmin" or "Samsung Galaxy Watch" or "mobile intervention*" or "mobile platform*" or "mobile solution*" or "health app*" or "mobile intervention" or "mobile" or "smartphone" or "iOS" or "Android" or "digital mental health intervention" or "mental health app" or "digital health platform" or "electronic health platform" or "eHealth platform" or "uHealth platform" or "ubiquitous health").mp. [mp=title, abstract, heading word, drug trade name, original title, device manufacturer, drug manufacturer, device trade name, keyword heading word, floating subheading word, candidate term word]	499011

4	("mental health" or "mental illness*" or "mental disorder*" or "psychological health" or "emotional health" or "mental wellness" or "mental well-being" or "psychological well-being" or "psychosocial well-being" or "anxi*" or "depress*" or "behavioural disorder*" or "cognitive health" or "psychological function*" or "burnout" or "mental health condition*" or "suicid*").mp. [mp=title, abstract, heading word, drug trade name, original title, device manufacturer, drug manufacturer, device trade name, keyword heading word, floating subheading word, candidate term word]	1972774

5	("literacy" or "knowledge" or "education" or "awareness" or "self-efficacy" or "help-seeking" or "help-seeking" or "support-seeking" or "support seeking" or "stigma*" or "competence" or "empowerment").mp. [mp=title, abstract, heading word, drug trade name, original title, device manufacturer, drug manufacturer, device trade name, keyword heading word, floating subheading word, candidate term word]	3242304

6	("RCT" or "randomized controlled trial*" or "randomised controlled trial*" or "randomized clinical trial*" or "randomised clinical trial*" or "controlled clinical trial*" or "random assignment" or "randomized study" or "randomised study" or "random allocation" or "controlled trial" or "clinical trial").mp. [mp=title, abstract, heading word, drug trade name, original title, device manufacturer, drug manufacturer, device trade name, keyword heading word, floating subheading word, candidate term word]	2591126

7	limit 6 to yr="2010 - 2025"	1755325

8	Mobile Applications/st, td [Standards, Trends]	0

9	3 or 8	499011

10	2 or 5	3242304

11	1 or 4	1972774

12	7 and 9 and 10 and 11	1911

**Web of Science**

Date of latest search: April 21, 2025

TS=("mobile health" OR "mHealth" OR "mobile application" OR "mobile app" OR "smartphone" OR "digital health" OR "mobile device" OR "mobile technology" OR "mobile phone" OR "tablet" OR "eHealth" OR "web app" OR "app" OR "apps" OR "gamification" OR "iPhone" OR "Android" OR "digital platform" OR "mobile tool" OR "digital application" OR "digital app" OR "smart device" OR "wearable device" OR "fitness tracker" OR "smartwatch" OR "Apple Watch" OR "Fitbit" OR "Garmin" OR "Samsung Galaxy Watch" OR "mobile intervention" OR "mobile platform" OR "mobile solution" OR "health app" OR "mobile" OR "iOS" OR "Android" OR "digital mental health intervention" OR "mental health app" OR "digital health platform" OR "electronic health platform" OR "eHealth platform" OR "uHealth platform" OR "ubiquitous health")

AND

TS=("mental health" OR "mental illness" OR "mental disorder" OR "psychological health" OR "emotional health" OR "mental wellness" OR "mental well-being" OR "psychological well-being" OR "psychosocial well-being" OR "anxiety" OR "depression" OR "behavioural disorder" OR "cognitive health" OR "psychological function" OR "burnout" OR "mental health condition" OR "suicide")

AND

TS=("literacy" OR "knowledge" OR "education" OR "awareness" OR "self-efficacy" OR "help-seeking" OR "help-seeking" OR "support-seeking" OR "support seeking" OR "stigma" OR "competence" OR "empowerment")

AND

TS=("RCT" OR "randomized controlled trial" OR "randomised controlled trial" OR "randomized clinical trial" OR "randomised clinical trial" OR "controlled clinical trial" OR "random assignment" OR "randomized study" OR "randomised study" OR "random allocation" OR "controlled trial" OR "clinical trial" OR "experiment" OR "intervention" OR "control group" OR "comparison group" OR "random selection")

AND

PY=(2010-2025)

**Cochrane Central Register of Controlled Trials (CENTRAL)**

Date of latest search: April 21, 2025

"mobile health" OR "mHealth" OR "mobile application" OR "mobile app" OR "smartphone" OR "digital health" OR "mobile device" OR "mobile technology" OR "mobile phone" OR "tablet" OR "eHealth" OR "web app" OR "app" OR "apps" OR "gamification" OR "iPhone" OR "Android" OR "digital platform" OR "mobile tool" OR "digital application" OR "digital app" OR "smart device" OR "wearable device" OR "fitness tracker" OR "smartwatch" OR "Apple Watch" OR "Fitbit" OR "Garmin" OR "Samsung Galaxy Watch" OR "mobile intervention" OR "mobile platform" OR "mobile solution" OR "health app" OR "mobile" OR "iOS" OR "Android" OR "digital mental health intervention" OR "mental health app" OR "digital health platform" OR "electronic health platform" OR "eHealth platform" OR "uHealth platform" OR "ubiquitous health" in Title Abstract Keyword AND "mental health" OR "mental illness" OR "mental disorder" OR "psychological health" OR "emotional health" OR "mental wellness" OR "mental well-being" OR "psychological well-being" OR "psychosocial well-being" OR "anxiety" OR "depression" OR "behavioural disorder" OR "cognitive health" OR "psychological function" OR "burnout" OR "mental health condition" OR "suicide" in Title Abstract Keyword AND "literacy" OR "knowledge" OR "education" OR "awareness" OR "self-efficacy" OR "help-seeking" OR "help-seeking" OR "support-seeking" OR "support seeking" OR "stigma" OR "competence" OR "empowerment" in Title Abstract Keyword AND "RCT" OR "randomized controlled trial" OR "randomised controlled trial" OR "randomized clinical trial" OR "randomised clinical trial" OR "controlled clinical trial" OR "random assignment" OR "randomized study" OR "randomised study" OR "random allocation" OR "controlled trial" OR "clinical trial" OR "experiment" OR "intervention" OR "control group" OR "comparison group" OR "random selection" in Title Abstract Keyword - with Publication Year from 2010 to 2025, in Trials with 'Public Health', 'Common Mental Disorders' in Cochrane Groups (Word variations have been searched)

# Table B. Summary of study characteristics, including study design, setting, and population^a^

| **Study ID** | **MHL Type** | **Measure** | **Randomization level** | **Country** | **Population** | **Intervention (s)** | **Age (mean (SD))** | **Follow-up time** | **ITT (Yes/No); Attrition Rate** |
| --- | --- | --- | --- | --- | --- | --- | --- | --- | --- |
| Bakker et al., 2018 | MHL | MHLQ | Individual | Australia | General population | Control (Waitlist), 64 | 33.6 (10.7) | 1 month | Yes; 55% |
|  |  |  |  |  |  | MoodKit, 56 | 33.8 (13.6) |  |  |
|  |  |  |  |  |  | MoodPrism, 56 | 36.1 (11.5) |  |  |
|  |  |  |  |  |  | MoodMission, 50 | 33.3 (12.8) |  |  |
| Birney et al., 2016 | Knowledge | Knowledge Scale | Individual | United States | Individuals with mild-moderate PHQ-9 symptoms) | Control (Web-based resources), 150 | 40.7 (11.2) | 2.5 months | Yes; 5% |
|  |  |  |  |  |  | MoodHacker, 150 | 40.6 (11.5) |  |  |
| Birrell et al., 2023 | Knowledge | Knowledge Scale | Cluster | Australia | Youth | Control (Standard Education Program), 78 | 15.2 (0.4) | 12 months | Yes, 92% |
|  |  |  |  |  |  | Mind your Mate Intervention, 88 | 15.2 (0.4) |  |  |
| Chu et al., 2019 | Knowledge, Help-Seeking | Mental Health Literacy Scale, Knowledge Scale | Individual | New Zealand | Youth | Control (No intervention), 112 | NA | 3 months | Yes; 9% |
|  |  |  |  |  |  | MyTeen, 109 | NA |  |  |
| García-Soriano et al., 2024 | MHL, Help-Seeking, Stigma | Attribution Questionnaire-27, GHSQ, MHLQ | Individual (Cross-over design) | Spain | General population | Control (delayed-use group), 56 | 40.4 (15.15) | 10 days | Yes; 71% |
|  |  |  |  |  |  | esTOCma, 56 | 39.5 (16.78) |  |  |
| Kerber et al., 2023 | MHL, Knowledge, Help-Seeking | MHLQ, AMPHSS, IASMHS | Individual | Germany | Adults with clinically relevant symptoms of internalizing disorders | Control (Waitlist), 211 | 38.2 (10.72) | 6 months | Yes; 50% |
|  |  |  |  |  |  | MindDoc, 307 | 38.5 (11.19) |  |  |
| King et al., 2023 | MHL, Help-Seeking | GHSQ, Suicide awareness questionnaire | Individual | Australia | Construction workers | Control (Face-to-face training), 575 | 33.1 (12.4) | 3 months | Yes; 68% |
|  |  |  |  |  |  | MATESmobile, 509 | 34.0 (12.5) |  |  |
| Kruzan et al., 2022 | Help-Seeking, Stigma | ATSPPH Scale, ISMI Scale | Individual | United States | Youth with current and chronic NSSI history | Control (Email-based psychoeducation materials), 64 | 20.61 (2.8) | 2 months | Yes; 40% |
|  |  |  |  |  |  | TalkLife, 67 | 20.0 (2.2) |  |  |
| Lam et al., 2024 | Knowledge, Help-Seeking, Stigma | Australian National MHL and Stigma Survey | Cluster | New Zealand | Office workers | Control (Waitlist), 227 | 42.8 (9.7) | Immediately after intervention | Yes; 48% |
|  |  |  |  |  |  | mHealth app, 229 |  |  |  |
| Lintvendt., 2011 | Knowledge | Treatment Depression Literacy Scale | Individual | Norway | University students | Control (Waitlist), 82 | 27.5 (7.5) | 2 months | Yes, 38% |
|  |  |  |  |  |  | MoodGYM, 81 | 28.8 (7.2) |  |  |
| O’Dea et al., 2020 | Help-seeking | GHSQ | Individual | Australia | General population | Control (Waitlist), 60 | 14.9 (0.9); 87%, 13% | 12 months | Yes, 40% |
|  |  |  |  |  |  | WeClick, 55 | 14.8 (0.9) |  |  |
| Röhr et al., 2021 | Stigma | Self-Stigma of Mental Illness Scale | Individual | Germany | Syrian refugees | Control (Psychoeducational resources), 63 | 33.7 (11.4) | 4 months | Yes, 13% |
|  |  |  |  |  |  | Sanadak, 53 | 33.0  (11.0) |  |  |
| Seo et al., 2022 | Knowledge, Help-Seeking | Parental Depression Literacy Scale, 25-item Social Support Scale | Individual | South Korea | Postpartum mothers with a score ≥ 9 on the Edinburgh Postpartum Depression Scale. | Control (Information Leaflet), 36 | 33.4 (4.5) | 5 months | Yes, 27% |
|  |  |  |  |  |  | Happy Mother App, 37 | 33.5 (3.3) |  |  |
| Tuijnman et al., 2022 | Help-Seeking, Stigma | GHSQ, Dutch Depression Stigma Scale | Cluster | Netherlands | General population | Control (No intervention), 80 | 13.4 (0.7) | 6 months | Yes; 10% |
|  |  |  |  |  |  | Moving Stories, 86 |  |  |  |

^a^Abbreviations: NA, Not available; MHL, Mental Health Literacy; MHLQ, Mental Health Literacy Questionnaire; GHSQ, General Help-Seeking Questionnaire; AMPHSS, Assessment of Mental Health Related Patient Empowerment and Self-Management-Skills questionnaire; ATSPPH, Attitudes Toward Seeking Professional Psychological Help Scale; ISMI, Internalized Stigma of Mental Illness Scale; ITT, Intention-to-Treat

^b^ Represents the mean age in years reported by studies

^c^ SD represents standard deviation

# Table C. Summary of Features Included in mHealth Interventions for MHL^a^

| **Study** | Cognitive-Behavioural Therapy | Gamification | Personalization | Mood-tracking | Educational Media |
| --- | --- | --- | --- | --- | --- |
| Bakker et al., 2018 (27) |  |  |  |  |  |
| Birney et al., 2016 (182) |  |  |  |  |  |
| Birrell et al., 2023 (181) |  |  |  |  |  |
| Chu et al., 2019 (180) |  |  |  |  |  |
| García-Soriano et al., 2024 (185) |  |  |  |  |  |
| Kerber et al., 2023 (187) |  |  |  |  |  |
| King et al., 2023 (188) |  |  |  |  |  |
| Kruzan et al., 2022 (30) |  |  |  |  |  |
| Lam et al., 2024 (186) |  |  |  |  |  |
| Lintvendt., 2011 (189) |  |  |  |  |  |
| O’Dea et al., 2020 (184) |  |  |  |  |  |
| Röhr et al., 2021 (183) |  |  |  |  |  |
| Seo et al., 2022 (31) |  |  |  |  |  |
| Tuijnman et al., 2022 (179) |  |  |  |  |  |

^a^Shaded cells represent the presence of the feature in the mHealth intervention

# Table D. Leave-one-out sensitivity analysis

| **Study excluded** | **SMD** | **Lower 95% CI** | **Upper 95% CI** | **I^2^** |
| --- | --- | --- | --- | --- |
| Bakker et al. 2018 | 0.149 | 0.046 | 0.252 | 88.164 |
| Birney et al. 2016 | 0.140 | 0.033 | 0.247 | 89.268 |
| Birrell et al. 2023 | 0.135 | 0.029 | 0.242 | 89.436 |
| Chu et al.  2019 | 0.118 | 0.013 | 0.224 | 88.617 |
| García-Soriano et al. 2024 | 0.104 | 0.007 | 0.201 | 78.888 |
| Kerber et al. 2023 | 0.116 | 0.011 | 0.222 | 87.546 |
| King et al. 2023 | 0.156 | 0.056 | 0.256 | 81.290 |
| Kruzan et al. 2022 | 0.138 | 0.029 | 0.247 | 89.359 |
| Lam et al. 2024 | 0.139 | 0.029 | 0.249 | 88.685 |
| Lintvedt et al. 2011 | 0.107 | 0.014 | 0.202 | 86.333 |
| O'Dea et al. 2020 | 0.131 | 0.025 | 0.236 | 89.390 |
| Röhr et al. 2021 | 0.141 | 0.033 | 0.249 | 89.165 |
| Seo et al. 2022 | 0.130 | 0.024 | 0.237 | 89.433 |
| Tuijnman et al. 2022 | 0.141 | 0.033 | 0.249 | 89.078 |

# Fig A. Traffic light plot showing results of the risk-of-bias assessment across studies

# Fig B. Traffic light plot showing results of the risk-of-bias assessment across studies

# Fig C. Forest plot of meta-analysis showing the effect of mHealth interventions on MHL among youth (aged 12–24 years)

# Fig D. Forest plot of meta-analysis showing the effect of mHealth interventions on MHL among adults (aged older than 25 years)

# Fig E. Forest plot of meta-analysis showing the effect of mHealth interventions on MHL among studies with short-term follow-up (≤3 months)

# Fig F. Forest plot of meta-analysis showing the effect of mHealth interventions on MHL among studies with long-term follow-up (>3 months)

# Fig G. Overall meta-analysis excluding studies with an active control group

# Fig H. Forest plot of overall meta-analysis excluding studies with high risk of bias

# Fig I. Forest plot of overall meta-analysis excluding studies with a niche participant population

# Fig J. Forest plot of overall meta-analysis excluding studies that used a cluster or cross-over experimental study design

# Fig K. Forest plot of overall meta-analysis excluding studies that involved participants with existing mental health symptoms

# Fig L. Funnel plot of overall meta-analysis assessing publication bias

**Formula 1: Weight of Individual studies**

$$w_{i}= \frac{1}{{{SE}_{i}}^{2}}$$

**Formula 2: Composite Effect Size (Hedge’s g)**

$$\mathrm{Composite}g_{i}= \frac{\sum(g_{i} \times w_{i})}{\sum w_{i}}$$

**Formula 3: Composite Mean (Control and Experimental)**

$${\mathrm{Composite}\bar{x}_{i}}_{Control}= \frac{\sum({Control \bar{x}}_{i}\times w_{i})}{\sum w_{i}}$$

$${\mathrm{Composite}\bar{x}_{i}}_{Experimental}= \frac{\sum({Experimental \bar{x}}_{i}\times w_{i})}{\sum w_{i}}$$

**Formula 4: Composite Standard Deviation (Control and Experimental)**

$${\mathrm{Composite}\sigma_{i}}_{Control}= \sqrt{\frac{{\sum w_{i} \times(Control {\sigma_{i}}^{2}(Control \bar{x}_{i} - {\mathrm{Composite}\bar{x}_{i}}_{Control})}^{2})}{\sum w_{i}}}$$

$${\mathrm{Composite}\sigma_{i}}_{Experimental}= \sqrt{\frac{{\sum w_{i} \times(Experimental {\sigma_{i}}^{2}\times({Experimental \bar{x}}_{i} - {\mathrm{Composite}\bar{x}_{i}}_{Experimental})}^{2})}{\sum w_{i}}}$$

Where $w_{i}$represents the weight of the study, intervention, or measure, relative to the other studies. The mean of the sample is represented by $\bar{x}_{i}$ and the standard deviation is represented by $\sigma_{i}$. The effect size measured as standardized mean difference (Hedge’s *g*), is represented by $g_{i}$.

**Fig M. Formulas for post-intervention summary statistics and handling unit-of-analysis issues**
